# Investigation of Start Block and Corresponding Influence for Grain Selection during Casting of Single-Crystal Superalloys

**DOI:** 10.3390/ma12101717

**Published:** 2019-05-27

**Authors:** Xintao Zhu, Fu Wang, Tobias Wittenzellner, Shuaipeng Zhang, Susanne Hemes, Michael Mathes, Dexin Ma, Andreas Bührig-Polaczek

**Affiliations:** 1 Foundry Institute, RWTH Aachen University, Intzestrasse 5, 52072 Aachen, Germany; zhudb8@gmail.com (X.Z.); t.wittenzellner@gi.rwth-aachen.de (T.W.); zspdeutsch66106@gmail.com (S.Z.); d.ma@gi.rwth-aachen.de (D.M.); sekretariat@gi.rwth-aachen.de (A.B.-P.); 2 State Key Laboratory for Manufacturing System Engineering, School of Mechanical Engineering, Xi’an Jiaotong University, Xi’an, Shaanxi 710049, China; 3 Access e.V., RWTH Aachen University, Intzestrasse 5, 52072 Aachen, Germany; s.hemes@access-technology.de (S.H.); m.mathes@access-technology.de (M.M.)

**Keywords:** Starter block, grain selection, selecting mechanism, Ni-based single crystal superalloys

## Abstract

To figure out the impact of the parameters of a starter block (the diameter D and height H) on grain selection and the selecting mechanism, a spiral selector was measured with optical microscopy (OM) and electron backscatter diffraction (EBSD) during the solidification of Ni-based single crystal (SX) superalloys. In this experiment, starter blocks with diameters of 8 mm, 10 mm, 15 mm, and 30 mm and a height of 30 mm were designed to find the best parameters. Recommendations for optimizing starter block geometry are provided.

## 1. Introduction

**Figure 1:**
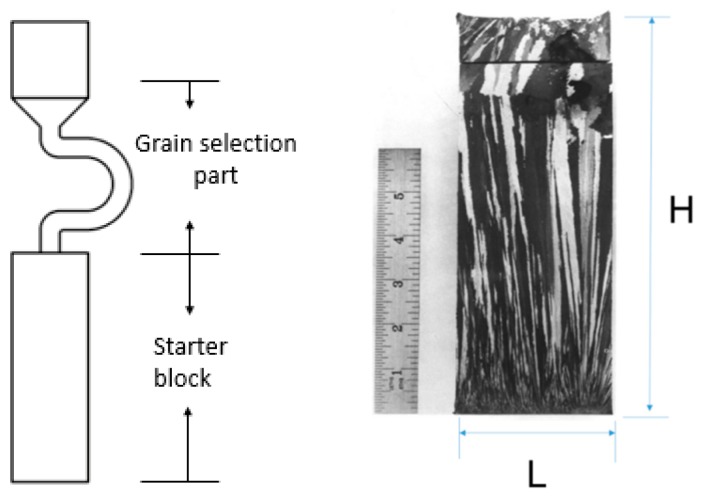
Illustration of the dimensions of the starter block.

Superalloys are a series of materials with high alloying strength and great surface stability with a Nickel or Cobalt matrix. They can be used at a temperature of above 600 °C and can withstand extremely high temperatures and pressures. In order to improve the temperature bearing capacity and high temperature mechanical properties of turbine blades, Ni-based superalloys have become the focus of research. Ni-based single-crystal (SX) superalloys such as CMSX-6, CMSX-4, and Mar M247LC are extensively used to make the blades of jet engines and industry gas turbines due to their excellent high temperature performance. In comparison with equiaxed (EQ) and directional solidified (DS) blades, the <001> crystallographic orientation of SX blades of Ni-based superalloys is parallel to the blades’ axis direction. For this reason, the creep performance can be optimized as long as the axis direction of SX blades approaches the <001> orientation. Nevertheless, a few deviations from <001> orientation can contribute to a poor creep property for the blades. Thus, an orientation of <001> close to the axial direction of SX blades has a great influence on the final performance of SX blades. To obtain a single-crystal structure, grain selectors are applied during directional solidification. In the process of SX casting, the grain selector is composed of a starter block and a grain selector [1,2,3]. The starter block optimizes the orientation of grains through competitive growth, and the selector ensures that only a single grain eventually survives. In general, most studies focus on the selector. However, the research on and design principles of the starter block have not been clearly presented [4,5]. In this paper, the proper parameters of the starter block are optimized with an experimental process. Prior studies [6,7,8,9] suggested that the function of the starter block is to optimize the grain orientation. Both the reduction of the diameter of the starter block and the increase in the height can improve the optimization of the <001> orientation so that the deflection angle of the grains is small and the final single crystal can be selected. However, not enough grains can enter a selector with a small diameter and a greater height, and the competition of different grain orientations may not be fully exerted, resulting in poor orientation results. Experimentally, starter blocks with diameters of 8 mm, 10 mm, 15 mm, and 30 mm and a height of 30 mm were designed to find the best parameters. Recommendations for optimizing the starter block geometry are also provided. The grain selecting mechanism in the starter block is mainly affected by two factors: one is competitive grain growth in the beginning, and the other is the blocking wall at the end. The improper design of the starter block would cause a bad grain angle and affect the <001> orientation of the single crystal structure. How starter block part is defined is shown in Figure 1.

## 2. Experimental Procedure

### 2.1. Casting Experiment 

The superalloy CM247LC was used in this study. Table 1 lists the chemical composition of CM247LC.

**Figure 2:**
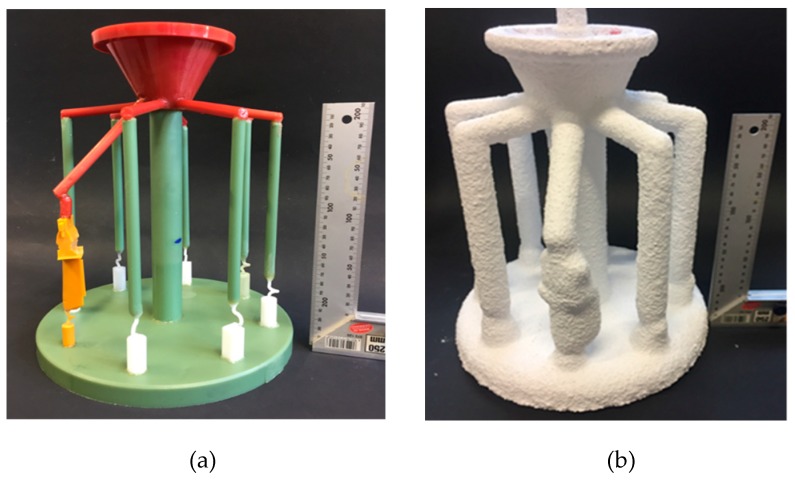
Images of (**a**) the wax model and (**b**) the shell mold.

To study the influence of the shape of the selector on grain selection, a wax bar composed of 2D grain selectors in different sizes and a cylindrical rod of 20 mm (D) × 150 mm (H) were injected as a whole. The parts were arranged around a center of gravity, forming a wax mold. Then, the wax group was immersed in a water-based ceramic mortar with different viscosities and painted with alumina mortar of different sizes. The whole process was repeated until the shell mold wall thickness reached 7~8 mm. After drying, the mold patterns were dewaxed. Thereafter, the mold was sintered to remove the remaining wax and the shell strength was increased. The wax mold and shell mold are shown in Figure 2. At last, the shell mold was mounted on a water-cooled copper cooler in a Bridgman furnace. In the process of investment casting, the shell mold was raised to a cylindrical heater and heated to 1470 °C. After equalizing the furnace temperature, the melted alloy was heated to 1500 °C and poured into the mold. The mold was withdrawn from the furnace for grain growth at a rate of 3 mm/min. As the temperature of the heater dropped below 300 °C, the vacuum was released, and then the mold was removed. Finally, the mold was knocked out, and the units and cluster were separated. 

### 2.2. Microstructural Characterization

After removing the ceramic debris, the units were etched to reveal the macrostructure using a 50% H_2_O_2_ + 50% HCl etchant. The selectors were then cut longitudinally and transversely and etched using 60 mL C_2_H_5_OH + 40 mL HCl + 2 g (CuCl_2_∙2H_2_O) etchant to show the dendrite structure in the selector. The structure of the dendrite is relatively complex, so we mainly used scanning electron microscopy to study the evolution trend and dynamic change of the dendrite structure.

## 3. Results and Discussion

In this study, the effect of the diameter of the starter block on grain selection was investigated by experimental research on starter blocks of different diameters. Table 2 lists the designed diameters of the starter block.

**Figure 3:**
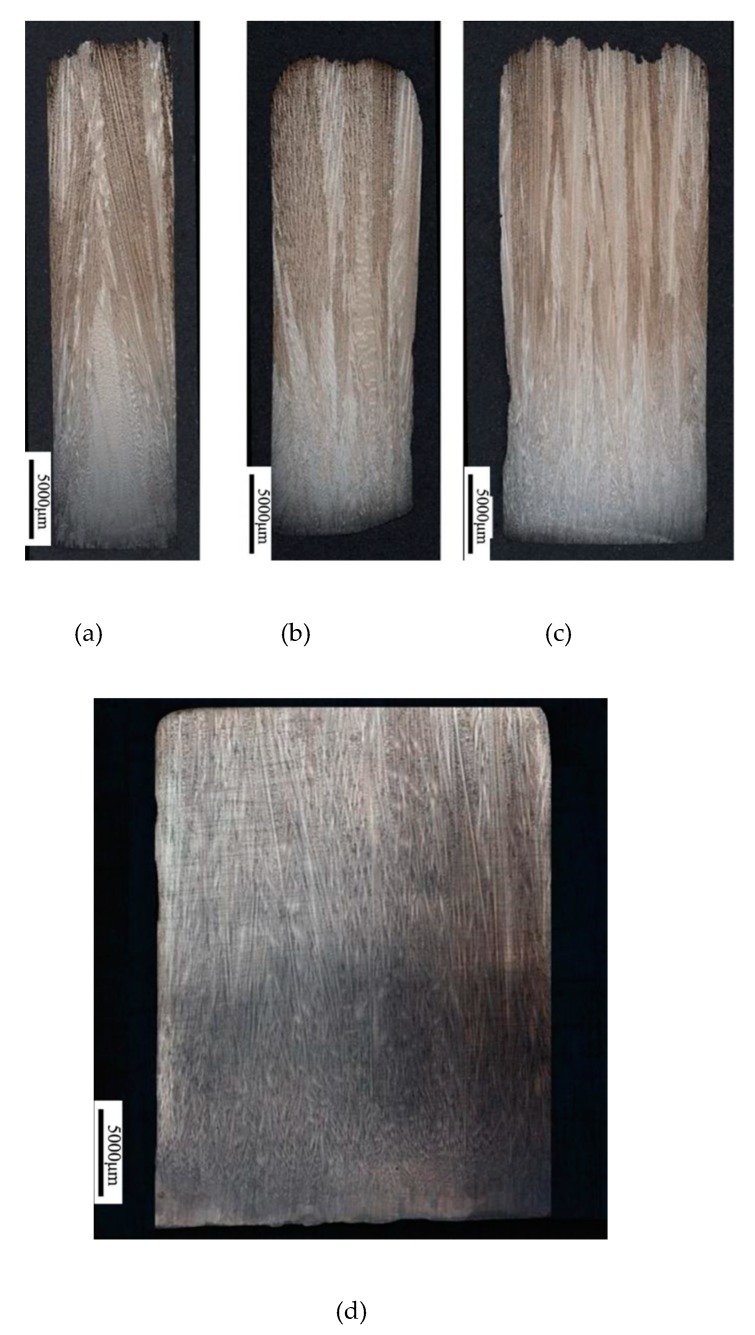
Metallographic photos of longitudinal sections of starters with different widths: (**a**) 8 mm, (**b**) 10 mm, (**c**) 15 mm, and (**d**) 30 mm.

Figure 3 shows the grain corrosion (optical microscopy, OM) of four starter blocks with different diameters at various height segments. According to the electron backscatter diffraction (EBSD) results, at the bottom of the starter block, the average <001> orientation deviation of the four different diameter grains is above 20 degrees, and the diameter has little effect on the chilling nucleation of the grains. When the heat transfer between the chill plate and the molten metal causes the temperature to reach below the liquidus of the alloy, the molten metal nucleates at the bottom of the seeding section to form an equiaxed crystal with different <001> orientations from each other.

However, as the solidification process progressed, the <001> orientation deviation of the grains in the four different starter blocks changed as the height increased. As shown in Figure 3, when the diameter of the starter block is 8 mm, the grain orientation deviation of the <001> direction is largely deflected for the thermal field of the shell wall influence due to the small diameter. When the diameter reaches 10 mm, the growth in the <001> direction is relatively uniform, and as the diameter becomes larger, the change in the convergence becomes slow. For the final grain, the <001> orientation with diameters of 10 mm and 30 mm did not change much. It can be seen that 10 mm is a critical condition for the size of the starter block diameter. Considering that as the diameter becomes larger, the metal used by the starter block becomes more numerous, and the diameter between 10 mm and 15 mm is the best design and application for the starter block.

**Figure 4:**
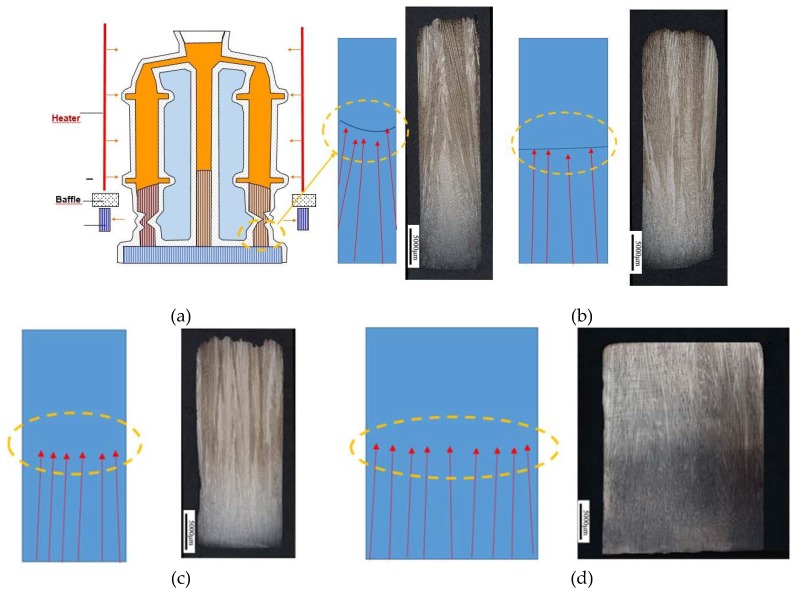
Competitive growth of longitudinal sections of starters with different width (**a**) 8 mm, (**b**) 10 mm, (**c**) 15 mm, and (**d**) 30 mm.

Figure 4 shows the longitudinal sections of four starter blocks. In all starter blocks, at the water-cooled chill area, the <001> orientations are randomly distributed and the number of grains is extremely large. The equiaxed grains at the bottom grow to form a column grains as the height increases. Meanwhile, the grains with large deviations are eliminated. Finally, only a small amount of grains with small deviation angles remain at the exit of the starter block. Figure 4 shows that with solidification, grains deviating from the <001> orientation are eliminated and the average orientation deviation becomes smaller and approaches 5°. The effect of height becomes insignificant after a 30 mm height is reached. 

**Figure 5:**
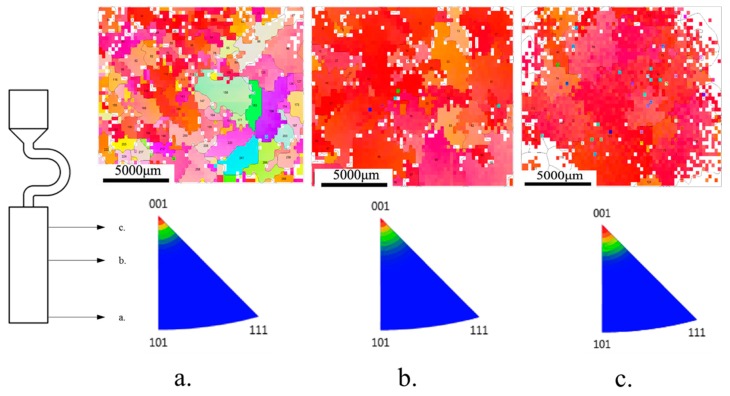
Diagrams and inverse pole figure at different heights with width 10 mm. (**a**) Height 2 mm; (**b**) Height 15 mm; and (**c**) Height 25 mm.

As shown in Figure 5 and Table 3, grains with <001> orientation (deflection less than 10°) grow preferentially, and the grains deviating from the <001> orientation would be eliminated gradually with the increase of distance from the bottom of the grain selector. 

**Figure 6:**
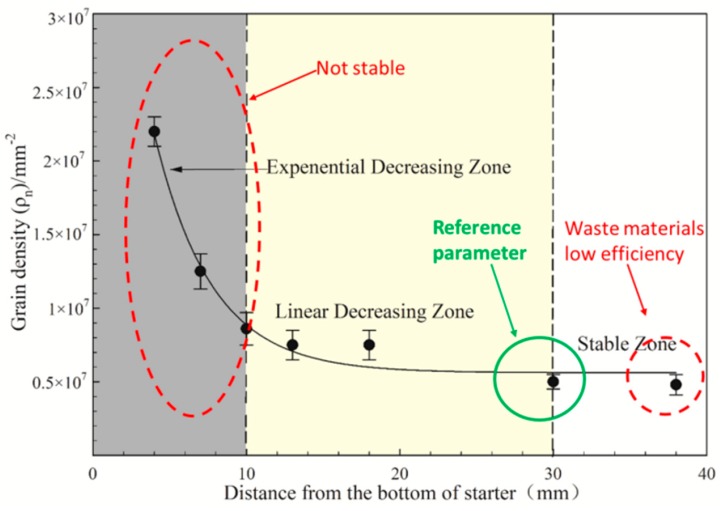
Grain density and average deviations between the <001> direction of grains and the direction of the heat flux as a function of distance from the bottom of the starter block.

Figure 6 shows that with the development of solidification, grains with worse orientation are eliminated, and average orientation deviation becomes smaller and approaches 5°. The effect of height becomes insignificant after a certain height. 

As shown in Figure 6, the number of grains in the range of 0–10 mm from the bottom is drastically reduced, and the rate of reduction from 10 mm to 30 mm is significantly slowed. Above 30mm, the number of grains remains stable.

**Figure 7:**
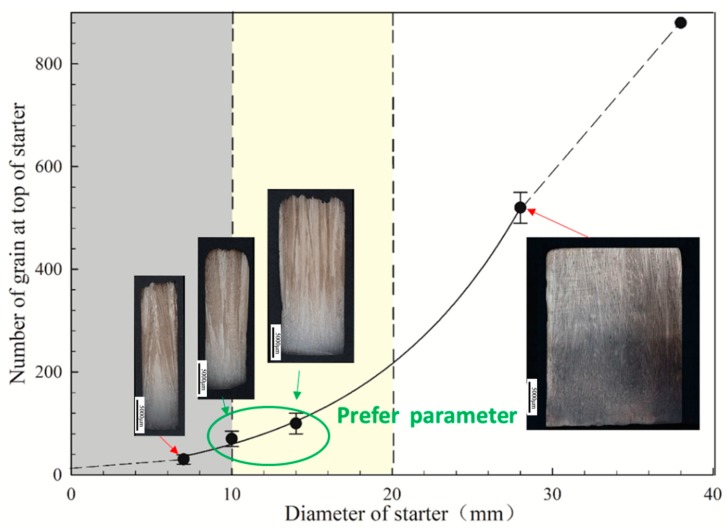
Grain number at the top of the starter as a function of width.

Figure 7 shows the number of grains at the top with different widths. Figure 4 shows that the change of widths has no effect on the average grain size and grain density. The decrements of width must then result in decreased grain numbers. To ensure that there is a proper amount of grains entering the grain selector wire, the width should neither be too large nor too small. 

When the starter block has a diameter of 8 mm, there were only about 26 grains in the top; when the diameter is 10 mm, 48 grains survived; and when the diameter is 30 mm, 520 grains were observed. Although reducing the diameter can improve the grain orientation optimization, when the diameter is too small, it will inevitably lead to the optimization of the orientation of the selector into the selector, which affects the grain selection efficiency and the choosing quality of the starter block. The starter block with a smaller diameter leads to not enough grains entering the selector, which makes the starter block unable to complete its optimizing function.

**Figure 8:**
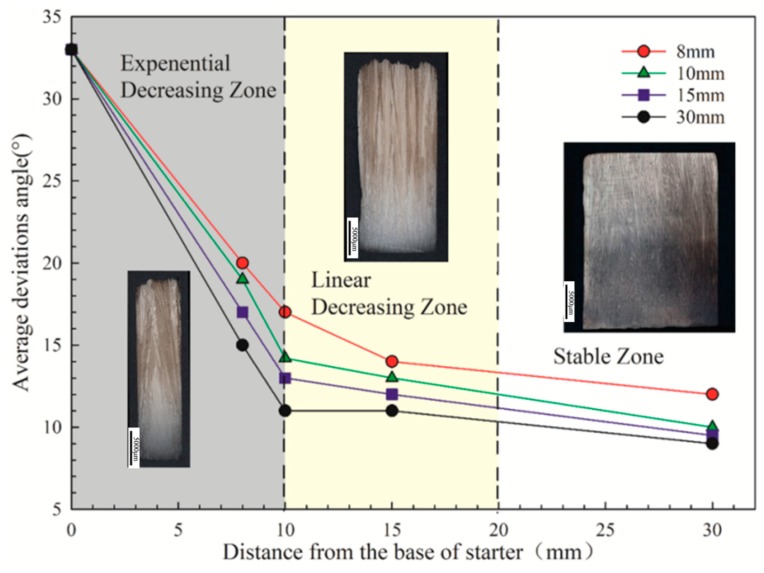
Average deviations between the <001> direction of grains and the direction of the heat flux at the top of the starter as a function of width.

**Figure 9:**
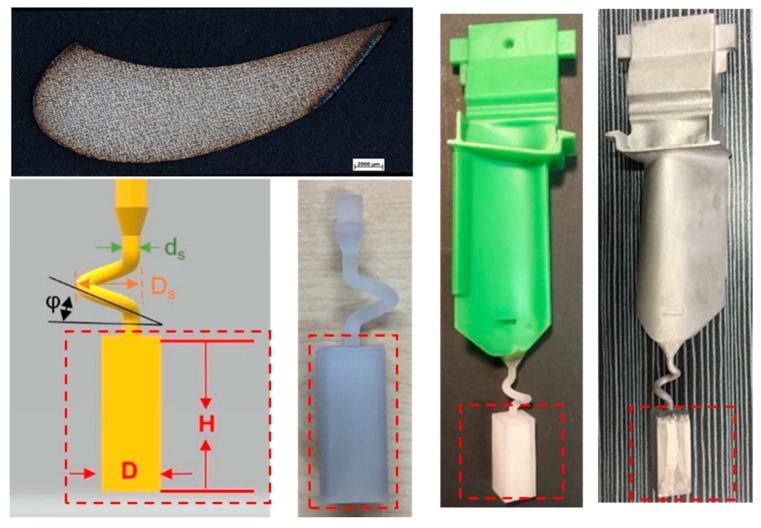
Schematic flow-process diagram of single crystal (SX) turbine blade investment casting with optimized starter block parameters (D = 10 mm and H = 30 mm).

Figure 8 shows that at the bottom, the deviation of the grains is above 30 degrees, and the diameter of the starter block has no influence on the chilling nucleation of the grains. When the heat transfer between the chill plate and the molten metal causes the temperature to reach below the liquidus of the alloy, the molten metal nucleates at the bottom of the starter block to form a layer of equiaxed grains of different <001> orientations, as shown in Figure 9. The average <001> orientation of the grains of the starter block of different diameters tends to be a fixed value. However, as the solidification progresses, the orientation optimization of different height segments is obviously changed. When the diameter of the starter block is too small (less than 10 mm), the liquidus and isotherms are unstable during solidification, resulting in an increase in the <001> orientation deviation. The starter block with a diameter of 15 mm or more shows little change in the <001> orientation. Considering the above, a starter block diameter of 10–15 mm is optimal.

However, the width should not always be as large as possible. A width of the starter block that is too large has negative effects on material costs. In addition, the number of grains at the exit of the starter block is so large that the selector cannot complete the singe crystal selection.

As shown in Figure 9, the optimized parameters of the starter block with a width of 10mm and a height of 30 mm was applied in a SX turbine blade casting with Mar M247LC. The grain selector was printed and the key geometry parameters were as listed below:

(1). Wax wire diameter (ds) = 3 mm;
(2). Pitch length (Ds) = 8 mm;
(3). Take-off angle (θ) = 40°
(4). Starter block height (H) = 30 mm
(5). Starter block width (D) = 10 mm

The single crystal structure of the SX turbine blade was measured by optical microscopy (OM) and the final performance of SX blades was verified successfully in application. 

## 4. Conclusions

The majority of the grain orientations in the whole selecting process was optimized in the starter block, and only a couple of grains with well-aligned orientations through competitive growth can enter the selector. The starter block is effective in optimizing in the <001> orientation, and the key parameter, i.e., the diameter, affects the efficiency of optimization.

(1) The main function of the starter block is to decrease the grain number and optimize the grain orientation.
(2) Grain selection in the starter block can be divided into two stages. The first stage is competitive growth between grains and the second stage is the blocking effect of the mold wall.
(3) The increase of width can enhance the optimization of the <001> orientation. It can be further explained by the effect of the mold wall on temperature distribution. However, a starter block with too large a width may arouse some problems in material costs. Combining the above; starter blocks with a width of 10 mm and a height of 30 mm should be adapted for all further studies.

## Figures and Tables

**Table 1:** The composition of superalloy CM247LC in wt%.

Elements	Al	Ti	Cr	Mo	Co	W	Ta	Hf	C	Ni
wt%	5.49	0.74	8.03	0.5	9.41	9.87	2.9	1.36	0.094	Bal.

**Table 2:** The designed parameters of the diameter of different starter blocks.

Case	1	2	3	5
Diameter/mm	8	10	15	30
Height/mm	30	30	30	30

**Table 3:** Grain numbers at different heights of grain selector.

Position	a	b	c
**Grain number**	69	35	21
